# Innovation through collaboration: Identifying opportunities to improve congenital anomalies surveillance in Canada

**DOI:** 10.17269/s41997-024-00949-8

**Published:** 2024-12-06

**Authors:** Yonabeth Nava de Escalante, Tanya Bedard, Cora Cole, Kitty Dang, Maya Jeyaraman, Kathryn Johnston, Qun Miao, Lauren Rickert

**Affiliations:** 1 Office of the Provincial Health Officer, BC Ministry of Health, Victoria, BC Canada; 2 Department of Family Practice, Faculty of Medicine, University of British Columbia, Vancouver, BC Canada; 3 Alberta Health Services, Alberta Congenital Anomalies Surveillance System, Calgary, AB Canada; 4 Nova Scotia Reproductive Care Program, Halifax, NS Canada; 5 Perinatal Surveillance Health PEI, Charlottetown, Prince Edward Island, Canada; 6 Government of the Northwest Territories, Department of Health and Social Services, Whitehorse, Northwest Territories, Canada; 7 Manitoba Health, Winnipeg, MB Canada; 8 New Brunswick Perinatal Health Program, Moncton, New Brunswick, Canada; 9 BORN Ontario, Ottawa, ON Canada; 10 Newfoundland and Labrador Health Services, St. John’s, Newfoundland and Labrador, Canada

**Keywords:** Congenital anomalies, Surveillance, Barriers, Facilitators, Intergovernmental collaboration, Population health, Anomalies congénitales, Surveillance, Obstacles, Facilitateurs, Collaboration innovante, Santé de la population

## Abstract

**Setting:**

The burden of congenital anomalies is a significant public health concern. In response to the World Health Organization’s recommendations, Canada developed and strengthened congenital anomalies surveillance to build capacity for prevention and optimal health outcomes. Historically, the Public Health Agency of Canada (PHAC) exclusively used hospital discharge data for the Canadian Congenital Anomalies Surveillance System (CCASS). A primary objective of the CCASS is to report prevalence, trends, and factors associated with congenital anomalies in Canada. However, the purpose of hospital discharge data is not for congenital anomalies surveillance; therefore, enhanced local data, which have more complete case ascertainment and additional data quality measures, are necessary.

**Intervention:**

Recognizing these significant limitations, PHAC, the provincial and territorial governments, physicians, public health practitioners, and academics collaborated on a project to enhance the CCASS with regional data and expertise. Subsequently, the Government of Canada InfoBase platform will use this enhanced dataset for national reporting.

**Outcomes:**

We developed standardized case definitions, a data submission form, and data quality tools, and surveyed programs to describe local congenital anomalies surveillance practice, and to identify barriers and facilitators that impact congenital anomalies surveillance efforts.

**Implications:**

This synergistic collaboration across jurisdictions, disciplines, and health care sectors is essential to support Canada’s enhanced congenital anomalies surveillance. We identified common themes on funding, operational requirements, data standardization, and legal and privacy considerations from the survey. These themes can be used to inform policy and decision-makers for sustainable congenital anomalies surveillance and to amplify the current momentum.

**Supplementary Information:**

The online version contains supplementary material available at 10.17269/s41997-024-00949-8.

## Résumé

**Contexte:**

Le fardeau des anomalies congénitales constitue une préoccupation majeure pour la santé publique. En réponse aux recommandations de l’Organisation mondiale de la santé, le Canada a développé et renforcé la surveillance des anomalies congénitales afin de renforcer les capacités en matière de prévention et d’optimiser les résultats en matière de santé. Historiquement, l’Agence de la santé publique du Canada (ASPC) utilisait exclusivement les données de congé hospitalier pour le Système canadien de surveillance des anomalies congénitales (SCSAC). Un objectif principal du SCSAC est de rapporter la prévalence, les tendances et les facteurs associés aux anomalies congénitales au Canada. Cependant, les données de congé hospitalier n’ont pas pour but de surveiller les anomalies congénitales; par conséquent, des données locales améliorées, qui présentent une vérification des cas plus complète des mesures supplémentaires de qualité des données, sont nécessaires.

**Intervention:**

Reconnaissant ces limitations importantes, l’ASPC, les gouvernements provinciaux et territoriaux, les médecins, les praticiens de la santé publique et les universitaires ont collaboré sur un projet visant à améliorer le SCSAC avec des données et des compétences régionales. Par la suite, la plateforme InfoBase du gouvernement du Canada utilisera cet ensemble de données amélioré pour les rapports nationaux.

**Résultats:**

Nous avons élaboré des définitions de cas normalisées, des formulaires de soumission de données, et des outils de qualité des données et avons enquêté auprès des programmes pour décrire les pratiques locales de surveillance des anomalies congénitales, et identifier les obstacles et facilitateurs qui influent sur les efforts de surveillance des anomalies congénitales.

**Incidences:**

Cette collaboration synergique entre les juridictions, les disciplines et les secteurs des soins de santé est essentielle pour soutenir la surveillance améliorée des anomalies congénitales au Canada. Nous avons identifié des thèmes communs liés au financement, aux exigences opérationnelles, à la normalisation des données, ainsi qu’aux considérations légales et de confidentialité à partir de l’enquête. Ces thèmes peuvent être utilisés pour informer les responsables politiques et décideurs en vue d’une surveillance durable des anomalies congénitales et pour amplifier l’élan actuel.

## Setting

Congenital anomalies (CA) are the leading cause of infant deaths in Canada, accounting for over 20% of these deaths (Public Health Agency of Canada, 2017; Statistics Canada, 2023). The World Health Organization (WHO) estimates that worldwide, 270,000 newborns die annually within 28 days of birth due to CA (WHO, 2020). Congenital anomalies also contribute to lifelong disabilities and significantly impact individuals, families, health care systems, and societies. The WHO, with the support of member states including Canada, committed to develop and strengthen CA surveillance systems to build capacity for prevention and optimal health outcomes (WHO, 2010).

The Canadian Congenital Anomalies Surveillance System (CCASS) was established to monitor and report on national prevalence, trends, and factors associated with CA (PHAC, 2010). This national surveillance system, managed by the Public Health Agency of Canada (PHAC), utilizes administrative hospital discharge data (Canadian Institute for Health Information, 2024; MED-ÉCHO, 2024) to ascertain CA cases. However, hospital discharge data have limited use in CA surveillance for the following reasons: (a) diagnostic codes are not routinely validated; (b) the ascertainment period is restricted to the first 30 days of life due to administrative reasons; and (c) the information pertaining to gestational age, maternal risk factors, and termination of pregnancies before 20 weeks of gestational age are incomplete (PHAC, 2013).

In Canada, provinces and territories administer and deliver health care within their jurisdiction. Thus, Canada’s health care system is based on ten provincial and three territorial health systems and provides opportunities to address the limitations of current national CA surveillance. In this paper, “provincial and territorial” are used interchangeably with “local”. Local surveillance systems have enhanced surveillance practices such as the inclusion of additional birth outcomes (e.g. termination of pregnancy under 20 weeks), multiple sources of ascertainment, verification of diagnostic codes, validated case definition algorithms, and local expertise, among others. Therefore, the inclusion of local CA data can significantly enhance and strengthen the quality of the national surveillance process (Boyd et al., 2005; Metcalfe et al., 2014; Nava de Escalante et al., 2023).

## Intervention

**Fig. 1:**
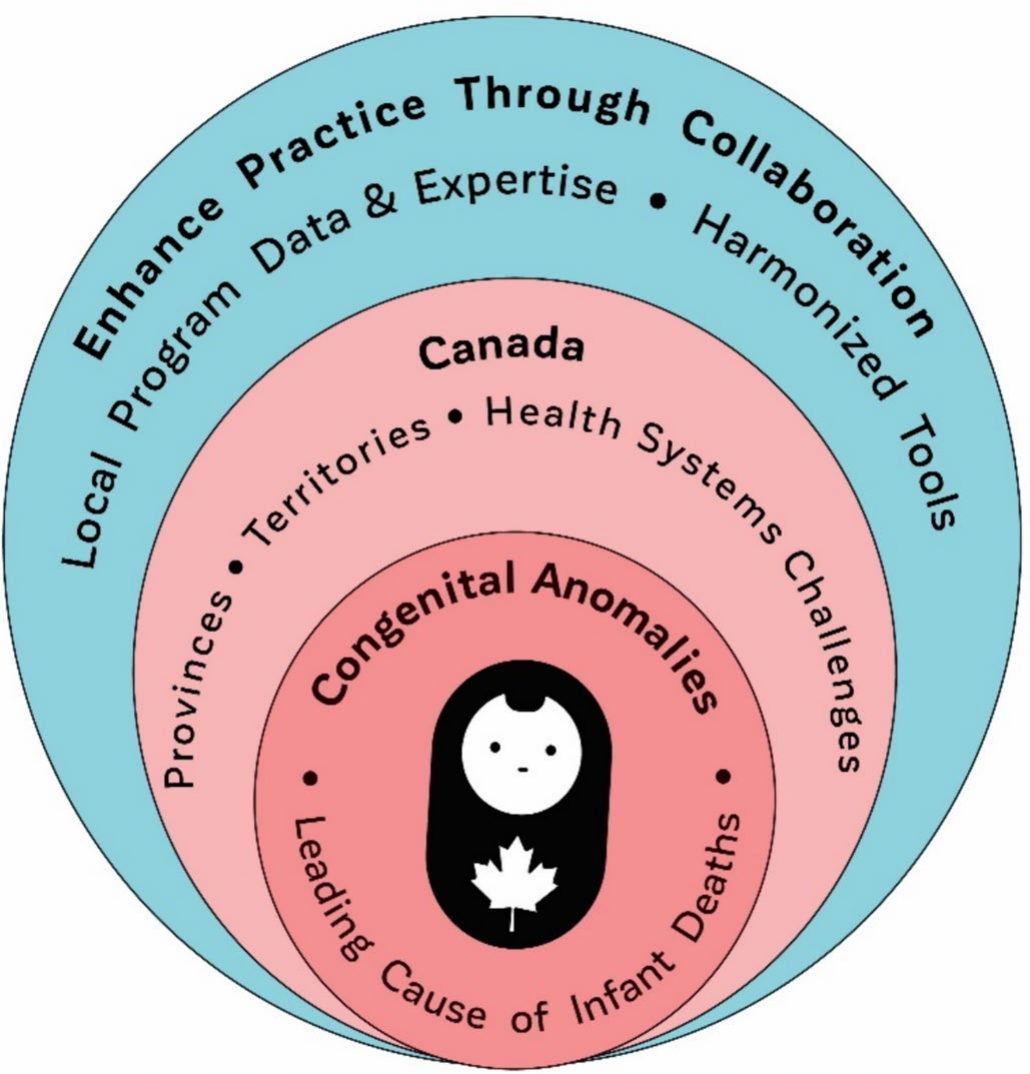
Enhance practice through collaboration: an initiative to enhance congenital anomalies surveillance in Canada

Recognizing the limitations of the CCASS data, the provincial and territorial governments, local CA programs, PHAC, physicians, public health practitioners, and academics collaborated to enhance the CCASS with regional data and expertise (Fig. 1). The main objective of this collaboration is to achieve standardized definitions regardless of the uniqueness of each CA program, to ensure sustainability and comparability between regions, and to agree on data quality standards required for effective national surveillance.

### Assessing feasibility

We used a modified version of the National Birth Defects Prevention Network’s State Birth Defects Surveillance Program Directory (National Birth Defects Prevention Network, 2019) to survey local CA programs and assess the feasibility for this collaboration in May 2023. Every program responded to the survey once via email or one-on-one interviews. This survey assessed program status with regard to surveillance methods (e.g. case definitions, data sources); ability to identify, record, and report CA cases; legal/privacy considerations; funding; possibility to expand ascertainment to capture maternal risk factors; and capacity for data sharing. This part of the survey provided an overview of the characteristics of each provincial and territorial CA surveillance system (Bedard et al., 2024). To support sustainability, we further analyzed additional information using thematic analysis and constant comparison methodology (Pope et al., 2000) to identify and report barriers and facilitators needed for continued support to the CCASS.

### Collaborative tool development

We promoted consistency in data reporting. We leveraged expertise from local programs to address issues related to case definitions, CA coding, standardization of variables, and data quality. We used resources previously disseminated and developed by the Canadian Congenital Anomalies Surveillance Network (the Network) to inform the development of tools to support standardized reporting and ensure consistent data quality for national reporting (International Clearinghouse for Birth Defects Surveillance and Research, 2024; Canadian Congenital Anomalies Surveillance Network, 2019).

### Fostering collaborative engagement across provinces and territories

Building upon insights from the feasibility assessment, we amplified the Network, an existing national network for CA surveillance, to serve as a platform for open communication. The Network facilitated monthly meetings, providing space for knowledge sharing, constructive discussion of common challenges, and potential solutions. Members worked collectively to establish standards for reporting, including time period for data collection, definitions (e.g. denominator, case definitions), and disaggregation of variables. We responded to emergent issues with ad hoc meetings. For example, a meeting was scheduled to discuss questions related to the program’s capacity to access information related to perinatal mental health. To foster accountability and transparency, we distributed actionable points within the group following each meeting. This ensured that all members were adequately informed and aligned with our goals and progress. Further, the Network convened annually to foster networking opportunities, nurture collaboration, present updates from local programs, and engage in open discussions around shared challenges, concerns, and strategies to enhance national surveillance of CAs. The meeting served as a forum for members to exchange insights and collectively strategize on best practices for CA surveillance.

## Outcomes

### Harmonization of tools

We used a previously created guideline for local CA surveillance (Canadian Congenital Anomaly Surveillance Network, 2019) and the International Clearinghouse for Birth Defects Surveillance and Research template (National Center on Birth Defects and Developmental Disabilities, 2019) to create a reporting template for the PHAC Infobase, a dataset primarily used for national-level reporting (ICBDSR, 2014). This reporting template has three sections. The first section provides surveillance definitions (e.g. data range, livebirth, stillbirths). The following sections collected information about the denominator and numerator, and yearly case reporting. Specifically, the denominator includes sources of ascertainment, number of total livebirths, and stillbirths. The numerator includes number of cases, disaggregated by birth outcome, maternal age, and infant sex (Supplemental Material A). To further support reporting, we also created a data quality assessment tool to help programs identify potential data quality issues (Supplemental Material B). Both supplemental materials are available for download for programs interested in replicating our work.

### Provision of local data

Eight provinces (British Columbia (BC), Alberta (AB), Manitoba (MB), Ontario (ON), Quebec (QC), New Brunswick (NB), Nova Scotia (NS), Newfoundland (NL)) and one territory (Nunavut (NU)) provided local aggregate data from their CA surveillance systems for future national reporting. These data will be used in the future PHAC InfoBase and will substitute the current health administrative data sources used (the Canadian Institute for Health Information – Discharge Abstract Database and Maintenance et exploitation des données pour l’étude de la clientèle hospitalière) (Public Health Agency of Canada, 2023). The future Infobase will be an interactive tool featuring three distinct tabs: Description, Data Tool, and Technical Appendix. The Description tab offers general information about CAs and it provides a comparison between administrative and local data for specific CAs. The Data Tool tab showcases local birth prevalence over a 5-year period and examines various factors associated with CAs, such as maternal age and infant sex. Finally, the Technical Appendix tab includes detailed information about data sources, methodologies, case definitions, and limitations. This updated online platform will be accessible to the public at https://health-infobase.canada.ca/congenital-anomalies/.

### Survey analysis

**Fig. 2:**
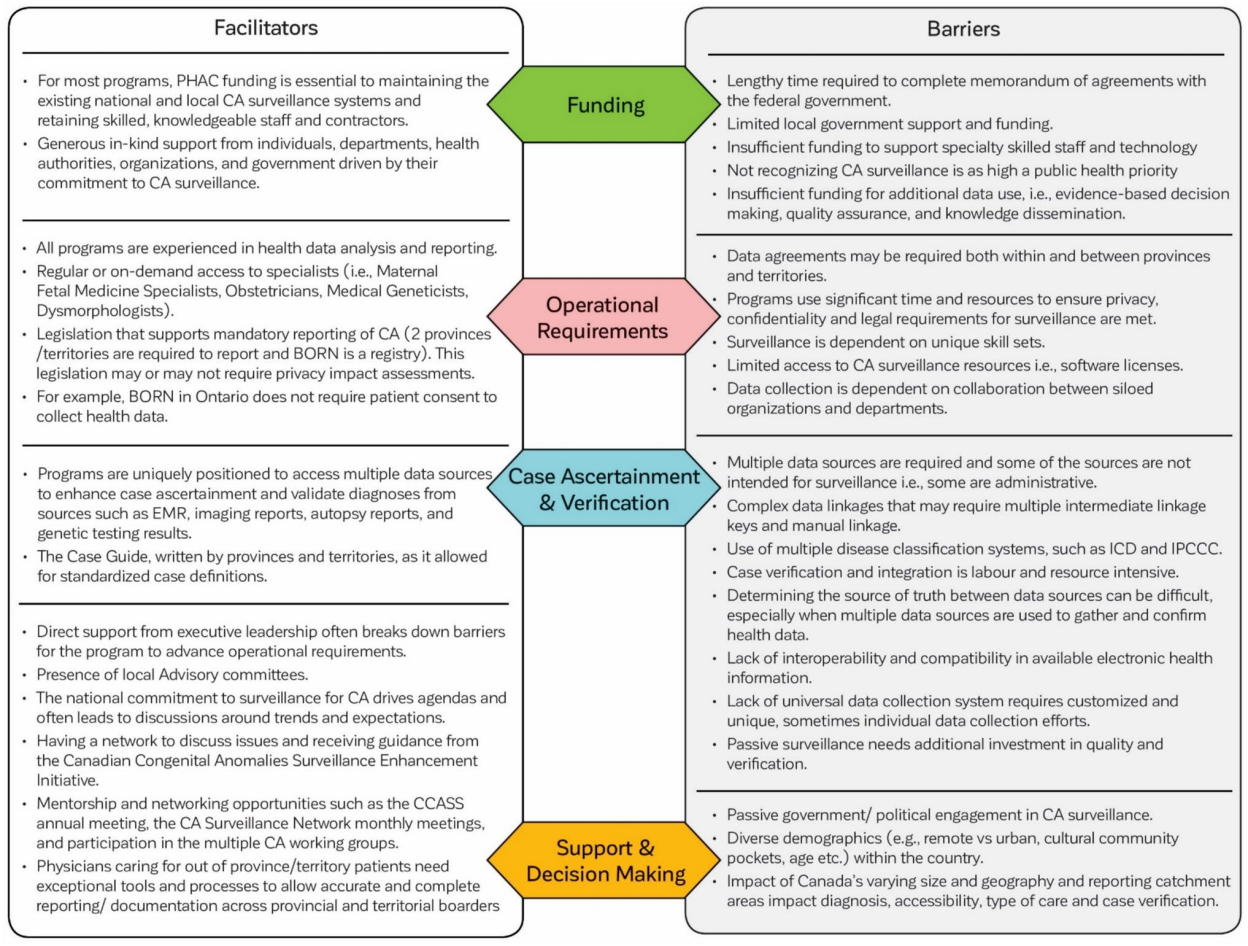
Barriers and facilitators for programs participating in the Canadian Congenital Anomalies Surveillance System

Twelve out of 13 provincial/territorial CA programs participated in the survey resulting in a completion rate of 92%. The survey identified four key areas: (1) funding, (2) operational requirements, (3) case ascertainment and verification, and (4) support and decision-making (see Fig. 2).

#### Funding

Funding from PHAC and/or supports (experts in the field, organizational departments, health authorities, and local governments) play a vital role in sustaining existing national and local CA surveillance systems. The significance of consistent and dedicated funding by the provincial and territorial governments to retain skilled specialist staff and support the cost of technology is critical. Additionally, improving the memorandum of agreement process with PHAC is essential for those who depend on PHAC funding. Programs identified potential efficiencies to CA surveillance by incorporating CA as part of the existing public health strategic plan.

#### Operational requirements

Facilitators supporting the operational requirements of CA surveillance systems include experience of specialized staff, regular access to experts, and legislation mandating the reporting of CA. However, there remain significant challenges in multiple areas. These include the lack of interjurisdictional data sharing agreements; the significant resources needed to adhere to privacy, and confidentiality requirements; the dependence on unique staff skill sets; and the impact of staff turnover.

#### Case ascertainment and verification

Programs are uniquely positioned to use multiple data sources for case ascertainment and verification. However, the linkage process for all these databases is complex and labour-intensive, most times requiring multiple intermediate linkage keys and manual work. Another barrier is the lack of interoperability and compatibility in available electronic health information, which adds to the complexity of case ascertainment and verification. Programs expressed the desire for a universal data collection system, which would reduce time and labour required for case ascertainment and verification.

#### Support and decision-making

Programs highlighted the importance and necessity to maintain support from the local executive leadership to champion CA surveillance programs and to advance operational requirements. The establishment and maintenance of provincial and territorial advisory committees provide multidisciplinary support and guidance to local programs. Participation in the monthly meetings of the CA Surveillance Network provides peer-to-peer surveillance support, mentorship, and expertise. Engaging in an annual day-long meeting to explore national opportunities for CA enhancement and address recurring data quality challenges serves as a key driver for advancing surveillance.

## Implications

We focused on fortifying national CA surveillance by integrating local data and expertise and ensuring a deep understanding of the common barriers and facilitators experienced by programs.

The future Infobase will provide an overview of national CA prevalence, and can also offer an insight into better estimates of regional variations and trends for participating jurisdictions. This will be particularly relevant for local programs that do not have a platform to report CA data. Not all provinces and territories were able to submit data for the future Infobase; however, most jurisdictions contributed to its planning through their participation in the CA Surveillance Network. Our vision is to expand this effort in the future and establish a national Infobase that encompasses CA data from all provinces and territories. We aim to create a comprehensive and unified platform that will contribute to the effectiveness and success of nationwide CA surveillance efforts.

Each program is at a different state of development and utilizes a different methodology of ascertainment. However, a standardized reporting template, with guidance from subject matter experts and more mature programs, allows for the collection of uniform data, essential for national reporting. 

We have learned and confirmed that data from local programs are substantially more comprehensive and valid than data from the CCASS. A comparison of the CCASS with a local CA surveillance system reported that rates of some CAs (e.g. hydrocephalus, lung hypoplasia) tended to be higher estimates than those from the local system because of diagnostic and coding issues, and a lack of validation for anomalies in CCASS (Wen et al., 2000). Additionally, the prevalence of common trisomies (e.g. Down syndrome, trisomy 18, and trisomy 13) is often substantially higher in local data than in data from the CCASS because of the inclusion of multiple data sources and birth outcomes in local data. These limitations were also observed when comparing CA data from administrative health systems with those from local CA surveillance systems (Bakker et al., 2023; Metcalfe et al., 2014).

Current program staff are committed, experienced, and trained in CA surveillance and data linkage methodologies. Yet, without sustainable funding, there is a risk of losing this expertise and corporate memory, which could contribute to staff turnover. The funding provided by PHAC is essential, as it fully funds or supplements local operational budgets.

Due to the wide variability in local policies and practices, CA surveillance is complex (Bakker et al., 2019). CA surveillance is further complicated by diagnoses that may change over time, such as prenatal versus postnatal diagnosis. Consequently, local programs often require input from specialists who are in high demand, including pediatric cardiologists, pediatric pathologists, medical geneticists, and maternal fetal medicine specialists. This increased demand emphasizes the need for local programs to retain highly trained staff.

Each program is responsible for case ascertainment in their own jurisdiction. Most programs only ascertain cases among their residents. Therefore, some CA are not being reported due to the lack of interjurisdictional data sharing agreements. For example, births with complex medical needs, such as spina bifida, may be sent to another province for delivery. These cases are not captured by the surveillance systems for the place of residence or delivery, potentially leaving these CA unreported. As a result, interjurisdictional data sharing agreements are needed to provide an accurate picture of certain anomalies. In some jurisdictions, these agreements require approval from multiple ministerial departments, leading to multi-year review processes. For some programs, data agreements within their own jurisdiction are also needed to access existing provincial or territorial databases. For instance, an internal data sharing agreement may be required for data to flow between a pediatric cardiology department and the local ministry of health, or between a fetal assessment unit and a provincial or territorial perinatal program. This is time-consuming and leads to reporting delays.

Full privacy impact assessments are required for almost all programs and are resource and time intensive. While these assessments identify and address privacy risks to ensure protection of health information, the process is not standardized across or within jurisdictions. This hinders the ability and need for CA surveillance systems to be agile to respond to new data needs. Any changes to data collection must undergo privacy review. Having legislation that supports the collection of identifiable information specifically for CA surveillance systems would improve this process. Precedent exists in British Columbia and Northwest Territories as they already have this legislation in place.

Further, complex data linkages and the lack of interoperability in electronic health information systems may limit the program’s capacity to produce timely data for reporting. In addition, the cost of data management and analytical software licenses, particularly with the loss of SAS government licenses, will impact access and the ability to run proven and established validation processes for several programs.

The current CA Surveillance Network became a dynamic, responsive way to share knowledge and common practices to foster a more comprehensive and informed approach to CA surveillance. The Network operates as a community of practice and allows for cross-disciplinary insight and the sharing of analytical resources and expertise. It also works as a channel to exchange relevant information concerning CA trends that may need to be investigated. The Network promotes an innovative approach to surveillance, as collaboration encourages a forward-thinking mindset, where programs feel more comfortable sharing issues and experiences, and exploring unconventional solutions. Younger CA surveillance programs benefit from the expertise and guidance from the more mature programs. The barriers and facilitators identified are important to navigate advocacy and set priorities for enhanced CA surveillance. Understanding common barriers and facilitators can guide resource investment and disinvestments within CA surveillance systems. Sustaining and continually evolving is crucial to harness synergies and prevent stagnation within the Network.

## Implications for policy and practice

What are the innovations in this policy or program?

- Our initiative focuses on enhancing the Canadian Congenital Anomalies Surveillance System with local data and expertise, as well as providing a better understanding of the common barriers and facilitators to CA surveillance across the country.
- We created tools for consistent reporting and enhancement of data quality.
- The CA Surveillance Network amplified communication and collaboration.
- Data collected and the synergy of expertise under this collaboration will help enhance national CA surveillance.
- Barriers and facilitators to CA surveillance can help inform federal and provincial governments’ decisions around investment and disinvestment in this area.

What are the burning research questions for this innovation?

- Is the risk of unstable funding worth the risk of a collapsing surveillance system and inability to monitor for CA?
- How can barriers be effectively addressed, and facilitators be promoted to ensure a more real-time and responsive congenital anomalies surveillance system?
- How can we continue to strengthen collaboration between the federal, provincial, and territorial governments, public health agencies, and research institutions to create a more integrated and holistic approach to congenital anomalies surveillance?

## Supplementary Information

Below is the link to the electronic supplementary material.

Supplementary file1 (XLSX 31 KB)

Supplementary file2 (XLSX 127 KB)

## Data Availability

Not applicable.
